# An *in situ* Gelling System Based on Methylcellulose and Tranilast Solid Nanoparticles Enhances Ocular Residence Time and Drug Absorption Into the Cornea and Conjunctiva

**DOI:** 10.3389/fbioe.2020.00764

**Published:** 2020-07-07

**Authors:** Noriaki Nagai, Misa Minami, Saori Deguchi, Hiroko Otake, Hiroshi Sasaki, Naoki Yamamoto

**Affiliations:** ^1^Faculty of Pharmacy, Kindai University, Osaka, Japan; ^2^Department of Ophthalmology, School of Medicine, Kanazawa Medical University, Ishikawa, Japan

**Keywords:** ophthalmic delivery, nanoparticles, *in situ* gelling system, tranilast, methylcellulose

## Abstract

We previously developed ophthalmic formulations containing tranilast nanopartaicles (ophthalmic TL-NPs formulations), and found them to show high uptake into ocular tissues. In this study, we aimed to design an *in situ* gel incorporating TL-NPs with 0.5–3% methylcellulose (MC, type SM-4) to ensure long residence time of the drug at the ocular surface. The ophthalmic TL-NPs formulations were prepared by the bead mill method, which yielded a mean particle size of ~93 nm with or without MC (0.5–3%). Although the dispersibility of TL particles in ophthalmic formulations increased with the MC content, the diffusion behavior of TL particles in the dispersion medium decreased with MC content. In an *in vivo* study using rats, the TL content in the lacrimal fluid was enhanced with MC content in the ophthalmic TL-NPs formulations, and the optimum amount of MC (0.5–1.5%) enhanced the TL content in the cornea and conjunctiva, and an anti-inflammatory effect of TL in rats instilled with ophthalmic TL-NPs formulations was observed. On the other hand, excessive MC (3%) prevented the corneal uptake of TL-NPs after instillation, and the anti-inflammation effect of TL was lower than that of ophthalmic TL-NPs formulations with optimum MC (0.5–1.5%). In conclusion, we found that gel formulations of TL-NPs with 0.5 and 1.5% MC provided a prolonged pre-corneal and pre-conjunctival contact time of TL, and resulted in higher TL contents in the cornea and conjunctiva following instillation in comparison with TL-NPs with or without 3% MC. This is probably due to the balance between the higher residence time and faster diffusion of TL-NPs on the ocular surface. These findings provide significant information that can be used to design further studies aimed at developing ophthalmic nanomedicines.

## Introduction

Eye drops can be applied easily as therapy for ocular diseases, and their share is over 70% in the ophthalmic field. On the other hand, drug residence is impeded by tear turnover, the corneal surface, reflex blinking and nasolacrimal drainage (Bhattacharjee et al., 2019) so that the absorption in the eyes is <5% of the applied dose (Choi and Kim, 2018). Thus, traditional eye drops cannot provide and maintain an adequate drug concentration in the corneal and conjunctival tissues, and frequent instillation is often needed to provide a sufficient therapeutic effect. However, frequent instillation enhances the drug concentration in the blood, and leads to poor patient compliance and systemic side effects. To resolve this conundrum and the shortcomings of traditional eye drops, the development of novel topical administration formulations and ophthalmic drug delivery systems, such as nanosuspensions, nanostructured lipid carriers, liposomes, preformed gels (Almeida et al., 2014), dendrimers ion triggered release (Zhu et al., 2018c), ionic resin based suspension, a pH-sensitive system for drug release (Zhu et al., 2018a, b), ointments, bioadhesive polymers (Silva et al., 2017; Grimaudo et al., 2019) have recently been proposed (Gan et al., 2009; Shen et al., 2009; Hagigit et al., 2010; Hironaka et al., 2011; Sun et al., 2015). In particular, nanotechnology has been expected to be applied in the ophthalmic field, and many researchers have provided novel nanomedicines and nanodevices (Sahoo and Labhasetwar, 2003). We also designed solid nanoparticles coated with 2-hydroxypropyl-β-cyclodextrin (HPβCD) (Nagai and Ito, 2014; Nagai et al., 2014a, 2017; Minami et al., 2020), and reported that the corneal toxicity of solid tranilast (TL) nanoparticles (TL-NPs) is lower than that of commercially available TL eye drops (TL-sol), and that nanoparticles show high drug adhesion to corneal tissue (Nagai et al., 2014b; Minami et al., 2020). These ophthalmic drug delivery systems containing solid nanoparticles are useful, although further improvements are necessary to prolong the drug residence time in the lacrimal fluid and to enhance the absorption into the ocular tissues after instillation for such systems to provide effective therapy for ocular diseases. Therefore, we attempted to develop a sustained release preparation containing TL-NPs.

In the ophthalmic field, *in situ* gelling systems exist as solids before instillation, but change to a gel on the ocular surface (after instillation). An *in situ* gelling system seems to be a good candidate for prolonging the drug residence time and enhancing absorption into the ocular surface (Wu et al., 2019; Yadav et al., 2019). Three systems, thermosensitive, ion-activated (Rupenthal et al., 2011) and pH-sensitive (Bawa et al., 2009), have been introduced, and the thermosensitive type systems widely used in comparison with other two types, since they involve less ocular stimulation (Kawase et al., 2010). Various polymers, such as methylcellulose (MC), alginic acid, gellan gum, chitosan, xyloglucan and pectin, can potentially be used for *in situ* gelling systems (Madan et al., 2009). MC, a water-soluble non-ionic cellulose ether that dissolves due to its weak physical crosslinks (Kang et al., 2008), and undergoes inverse thermal gelling to form a physically crosslinked hydrogel at physiological temperatures, is frequently used as a gelling agent (Huang et al., 2016). In this study, we designed extended-release ocular *in situ* gelling systems by combining MC and TL-NPs [ophthalmic *in situ* gel (ISG)-formulations with TL-NPs]. In addition, we investigated the preventive effect of ophthalmic ISG-formulations with TL-NPs on inflammatory mediators, such as nitric oxide (NO) and tumor necrosis factor-α (TNF-α), in lipopolysaccharide (LPS) induced-rat conjunctival inflammation.

## Materials and Methods

### Animals

Male Wistar rats aged 7 weeks were purchased from Kiwa Laboratory Animals Co., Ltd. (Wakayama, Japan). Japanese albino rabbits (∼2.7 kg) were used to investigate the corneal toxicity of ophthalmic formulations. All animal experiments were performed in accordance with the guidelines of ARVO and Kindai University, and were approved on 1 April 2013 (project identification code KAPS-25-003) by the pharmacy committee for animal research of Kindai University. Inflammation in conjunctiva was induced by the injection (30 μL) of 0.2 mg/mL LPS in saline into the upper palpebral conjunctiva. After 30 min, traditional and ophthalmic TL-NPs formulations were instilled, and the eyes were kept open for 1 min after instillation to prevent washing out.

### Chemicals

TL powder and commercially available TL eye drops (TL-sol) were gifts from Kissei Pharmaceutical Co., Ltd.; 2-Hydroxypropyl-β-cyclodextrin (HPβCD) and type SM-4 methylcellulose (MC) were obtained from Nihon Shokuhin Kako Co., Ltd. (Tokyo, Japan) and Shin-Etsu Chemical Co., Ltd. (Tokyo, Japan), respectively. Benzalkonium chloride was purchased from Kanto Chemical Co., Inc. (Tokyo, Japan), and mannitol (D-mannitol), ethyl p-hydroxybenzoate and isoflurane were provided by Wako Pure Chemical Industries, Ltd. (Osaka, Japan). All other chemicals used were of the highest purity commercially available.

### Preparation of Ophthalmic Formulations Containing TL-NPs (Ophthalmic TL-NPs Formulations)

Table 1 shows the composition of the ophthalmic TL-NPs formulations (nTL, nTL-LMC, nTL-MMC, and nTL-HMC) prepared by using the bead mill method (Nagai and Ito, 2014; Nagai et al., 2014b, 2017; Minami et al., 2020). TL powder was dispersed in a purified water containing mannitol, benzalkonium chloride, and HPβCD, added into 1.5 mL tubes with 0.1 mm zirconia beads, and subjected to the Bead Smash 12 (Wakenyaku Co. Ltd, Kyoto, Japan). Bead mill treatment was performed under the following conditions: 5,500 rpm for 30 s × 10 times at 4°C. After bead mill treatment, MC was added, and the pH was adjusted to 6.5 (ophthalmic ISG-formulations with TL-NPs). TL concentrations were measured by the following HPLC method. Samples (10 μL) were diluted in 100 μL methanol containing 3 mg/L ethyl p-hydroxybenzoate (internal standard), mixed, and injected into a Shimadzu HPLC LC-20AT system (Shimadzu Corp. Kyoto, Japan) with an Inertsil ODS-3^®^ column (GL Science Co., Inc., Tokyo, Japan). The column was eluted at 35°C with 50 mM ammonium acetate and acetonitrile (80:20) at a flow rate of 0.25 mL/min. TL was detected at 230 nm.

**TABLE 1:** Composition of ophthalmic TL-NPs formulations.

Formulation	TL	MC	BAC	Mannitol	HPβCD	Purified water ad.	Treatment
nTL	0.5 g	–	0.001 g	0.1 g	5 g	100 g	Bead mill
nTL-LMC	0.5 g	0.5 g	0.001 g	0.1 g	5 g	100 g	Bead mill
nTL-MMC	0.5 g	1.5 g	0.001 g	0.1 g	5 g	100 g	Bead mill
nTL-HMC	0.5 g	3.0 g	0.001 g	0.1 g	5 g	100 g	Bead mill

### Characteristics of the Ophthalmic TL-NPs Formulations

The particle size of the TL powder was determined by a laser diffraction particle size analyzer Shimadzu SALD-7100 (Shimadzu Corp., Kyoto, Japan) with the refractive index set at 1.60–0.10i. Particle size and number in the TL-NPs formulations were measured by a Dynamic Light Scattering QuantumDesign NANOSIGHT LM10 (QuantumDesign Japan, Tokyo, Japan) for 60 s at 405 nm. The viscosities at 20 and 37°C were analyzed by an Anton Paar MCR302 attached to a CP50-1 (Anton Paar Japan K.K, Tokyo, Japan). The measurement was performed 10 times, and the mean was used in this study. The measurement conditions were as follows: measurement time 2 s, interval 1 s, shear rate 90–100 rpm/s. The atomic force microscopic (AFM) image created by combining a phase and height image was provided by a Shimadzu SPM-9700 (Shimadzu Corp., Kyoto, Japan). A Nihon Rufuto micro-electrophoresis zeta potential analyzer model 502 was used to determine the zeta potential (Nihon Rufuto Co., Ltd., Tokyo, Japan), and the crystal form was obtained using a Rigaku MiniFlex II (Rigaku Co., Tokyo, Japan). The samples for powder X-ray diffraction (XRD) were lyophilized and pulverized with the diffraction angles set from 5° to 90°, and measured at a scanning rate of 10°/min. TL solubility was measured using a Beckman Optima^TM^ MAX-XP Ultracentrifuge (Beckman coulter, Osaka, Japan) and HPLC method. For the measurement of solubility, non-solubilized TL-NPs were removed by centrifugation at 100,000 g, and the TL contents in the supernatants were measured. To measure the dispersibility of TL-NPs, ophthalmic TL-NPS formulations were stored for 3 months at 20°C, and samples were taken from 5 mm under the surface over time. The TL contents of the samples were determined by the HPLC method described above.

### Measurement of Ophthalmic TL-NPs Formulation Diffusion

A methacrylate cell equipped with a 0.22 μm-pore membrane filter was used (Nagai et al., 2014a; Minami et al., 2020). One side of the cell (donor chamber) was filled with an ophthalmic TL-NPs formulation, and the other side (reservoir chamber) was filled with saline. The experiments were performed at 37°C. Samples (50 μL) were taken from the reservoir chamber over time, and replaced with the same volume of buffer. The TL contents in the samples were measured by the HPLC method described above.

### Measurement of TL Contents in Lacrimal Fluid, Blood, Cornea, and Conjunctiva

Rats were instilled with ophthalmic TL-NPs formulations, and euthanized by injection of a lethal dose of sodium pentobarbital 30, 60, and 150 min after instillation. The lacrimal fluid was collected using schirmer tear test strips. Blood was collected from the vena cava, and centrifuged to provide serum. The corneas and conjunctiva were excised. TL was extracted from the lacrimal fluid in the schirmer tear test strips, blood (serum), cornea and conjunctiva in methanol, and centrifuged at 20,400 g for 20 min at 4°C. The supernatants were used as samples. TL contents were measured by the HPLC method described above. Protein levels in the samples were determined using a Bio-Rad Protein Assay Kit (BioRad Laboratories, Hercules, CA, United States), and the TL levels in the corneas and conjunctiva are expressed as nmol/mg protein.

### Measurement of Corneal Toxicity

Ophthalmic TL-NPs formulations (30 μL) were repetitive instilled to rabbits twice a day (9:00 and 19:00) for 1 month. After that, 30 μL of 1% fluorescein was instilled to stain the wound area, and the wound area were monitored by a Topcon TRC-50X (Topcon, Tokyo, Japan).

### Measurement of Vessel Leakage in Inflammation Using Evans Blue (EB)

EB was used to monitor vascular protein leakage. The EB (10 mg/kg) was injected to femoral vein 5 min before the injection of LPS. When the experiment was finished (150 min after the instillation of ophthalmic TL-NPs formulations), the rats were euthanized by injecting a lethal dose of pentobarbital, the blood was removed by perfusion with cold saline, and the conjunctiva were excised. The conjuntival tissues were homogenized in 1M KOH, and incubated for 24 h at 37°C. After that, the EB was extracted by incubation in 0.2 M phosphoric acid and acetone (5:13) for 2 h, after which the samples were centrifuged at 400 g for 15 min at 4°C, and the supernatants were used for measurements. EB exudation was determined from the absorbance at 620 nm, and is expressed as Abs/g weight of conjunctival tissue.

### Measurement of NO Levels

The conjunctiva were excised from rats after euthanasia were homogenized in saline on ice, and centrifuged at 20,400 g for 20 min at 4°C. The supernatants were filtrated by a concentric microdialysis probe (A-1-20-05, Eicom, Kyoto, Japan), and perfused to Eicom ENO-20 (Eicom, Kyoto, Japan) with Ringer’s solution at a constant flow rate of 2 μL/min. The samples were mixed Griess reagent in ENO-20, and the NO_2_^–^ and NO_3_^–^ levels were detected at 540 nm. In this study, NO amounts represent the total NO metabolite level, which is the sum of the NO_2_^–^ and NO_3_^–^ levels.

### Measurement of TNF-α Levels

Rats were euthanized by injecting a lethal dose of pentobarbital, and the conjunctiva were excised and homogenized in saline on ice. The homogenates were centrifuged at 20,400 g for 20 min at 4°C, and the supernatants were used for the measurement of TNF-α levels. TNF-α levels were measured using a Rat TNF-α Quantikine ELISA Kit (Bio-techne, Seattle, WA, United States) according to the manufacturer’s instructions. The TNF-α levels are expressed as pg/mg protein.

### Statistical Analysis

A minimum *P*-value of 0.05 was chosen as the significance level (*P* < 0.05), and Student’s *t*-test and one-way analysis of variance (ANOVA) followed by Dunnett’s multiple comparison were used for statistical analysis of groups. The sample numbers (*n*) are shown in the figure legends, and the data are expressed as mean ± standard error of the mean (SEM).

## Results

### Formulation and Evaluation of TL-NPs for Topical Ophthalmic Delivery

**FIGURE 1:**
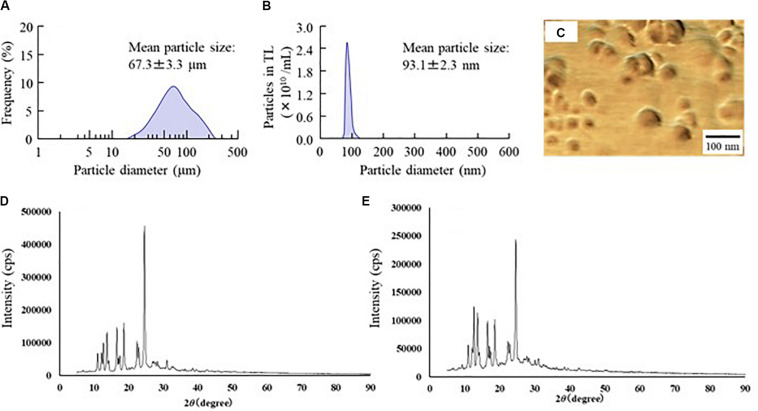
Changes in particle size frequency and crystal structure of TL before and after bead mill treatment. **(A,B)** Particle size frequencies of TL before **(A)** and after **(B)** bead mill treatment. **(C)** AFM image of TL following bead mill treatment. **(D,E)** XRD patterns of TL before and after bead mill treatment. Bead mill treatment decreased the particle size to a mean of 93.1 ± 2.3 nm. The crystal structures were similar for TL both before and after bead mill treatment.

**FIGURE 2:**
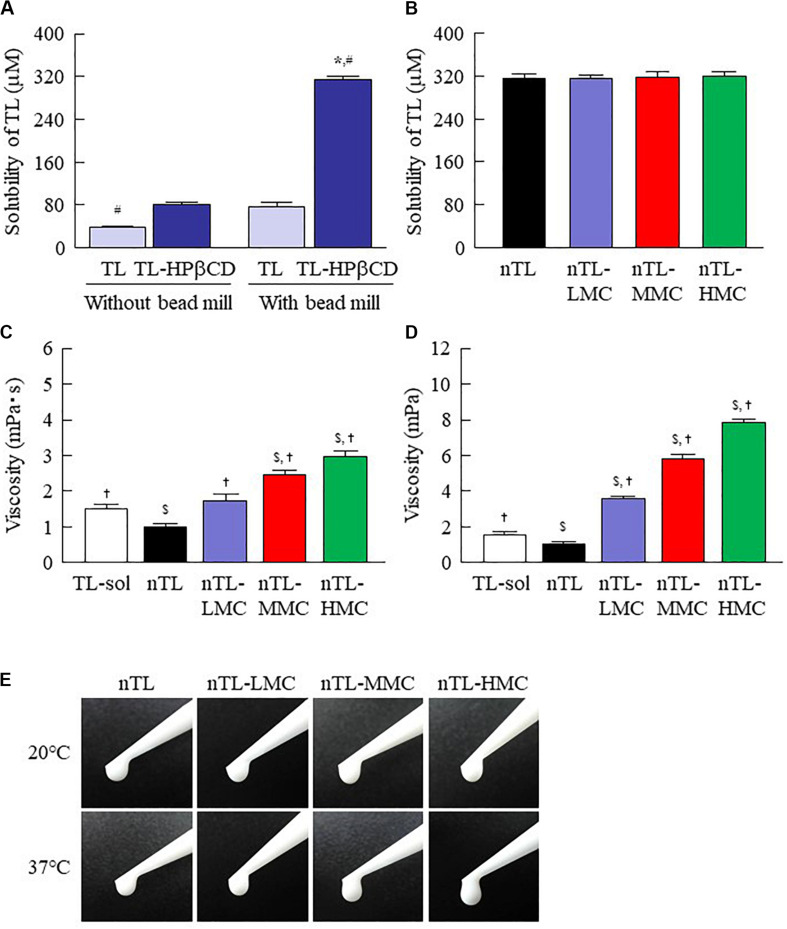
Solubility and viscosity of TL in ophthalmic TL-NPs formulations. **(A)** Effect of HPβCD on the solubility of TL with or without bead mill treatment. **(B)** Solubility of TL in ophthalmic TL-NPs formulations. **(C,D)** Viscosity of TL in ophthalmic TL-NPs formulations at 20°C **(C)** and 37°C **(D)**. **(E)** Pictures in ophthalmic TL-NPs formulations at 20°C and 37°C. *n* = 10. The compositions of the ophthalmic TL-NPs formulations are shown in Table 1. *P < 0.05 vs. TL for each category. ^#^*P* < 0.05 vs. TL-HPβCD without bead mill for each category. ^$^*P* < 0.05 vs. TL-sol for each category. ^†^*P* < 0.05 vs. nTL for each category. The TL/HPβCD inclusion complex was enhanced by bead mill treatment, and the solubility was increased. Although the addition of MC enhanced viscosity, the solubility of TL was not affected by the MC content.

**FIGURE 3:**
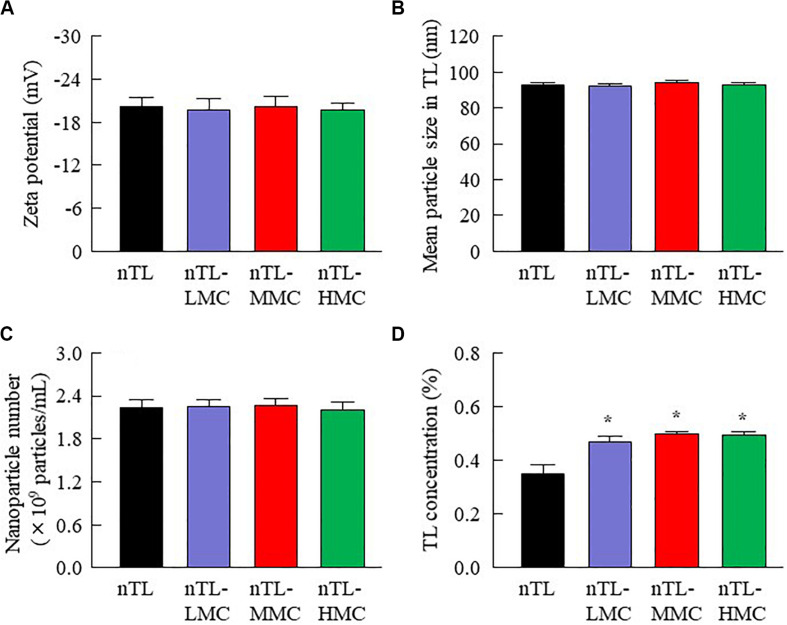
Zeta potential, particle size, nanoparticle number and dispersibility of ophthalmic TL-NPs formulations. **(A–D)** Effect of MC content on the zeta potential **(A)**, particle size **(B)**, nanoparticle number **(C)**, and dispersibility **(D)** of TL in ophthalmic TL-NPs formulations. *n* = 8. The compositions of the ophthalmic TL-NPs formulations are shown in Table 1. *P < 0.05 vs. nTL for each category. The addition of MC had no effect on the zeta potential, particle size or nanoparticle number of TL in ophthalmic TL-NPs formulations. On the other hand, the dispersibility of TL-NPs was enhanced with MC content.

First, we designed formulations containing TL-NPs using the bead mill method, and evaluated their characteristic parameters. Figure 1 shows the state of TL before and after bead mill treatment. The particles in TL powder prior to bead mill treatment show a wide size distribution of 15–250 μm (Figure 1A). The particle size and distribution were both decreased by bead mill treatment to a range of 80–120 nm (Figures 1B,C). Furthermore, bead mill treatment did not affect the crystal structure of TL since the XRD pattern of TL before and after the bead mill was the same (Figures 1D,E). It is known that drug solubility is increased by nanoparticulation and the addition of HPβCD. In this study, the solubility of TL-NPs was higher than of the TL powder. The TL/HPβCD inclusion complex was also increased by bead mill treatment, and the solubility of TL-NPs with HPβCD was 8.52–fold that of TL powder without HPβCD (Figure 2A). On the other hand, the solubility was not changed by the addition of MC (Figure 2B). Solubilized TL accounts for ~2% of the total TL in all formulations (the remaining ~98% exists as solid TL nanoparticles). Figures 2C,D show the effect of MC addition on the viscosity of the ophthalmic TL-NPs formulations. The viscosity of TL-NPs without MC (nTL) was lower than that of commercially available TL eye drops (TL-sol). The viscosity of the ophthalmic ISG-formulations with TL-NPs (nTL-LMC, nTL-MMC, and nTL-HMC) is enhanced with increasing MC content (Figure 2C). Moreover, the ophthalmic ISG-formulations with TL-NPs were gelled at 37°C, and the viscosities of nTL-LMC, nTL-MMC, and nTL-HMC were 3. 55-, 5. 71-, 7.71-fold greater than that of nTL at 37°C (Figures 2D,E). Figure 3 shows the effect of MC addition on the zeta potential, particle size, nanoparticle number, and dispersibility of ophthalmic TL-NPs formulations. The enhanced viscosity caused by MC increased the dispersibility; no aggregation or precipitation of TL was observed in the ophthalmic ISG-formulations with TL-NPs (nTL-LMC, nTL-MMC, and nTL-HMC) (Figure 3D). In the contrast to the results for viscosity and TL dispersibility, the solubility, zeta potential, particle size, and nanoparticle number of the ophthalmic TL-NPs formulations were not affected by the MC content (Figures 3A–C).

### Drug Behavior in Rats Instilled With Ophthalmic TL-NPs Formulations

**FIGURE 4:**
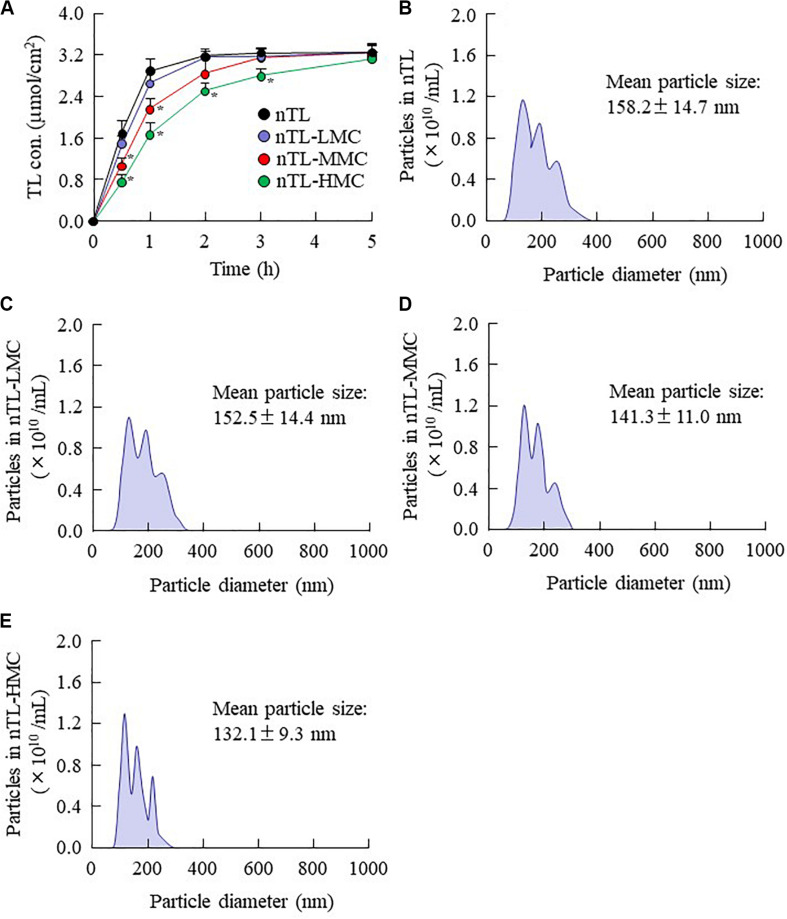
Diffusion of TL-NPs in ophthalmic TL-NPs formulations through a methacrylate cell. **(A)** Diffusion behavior of TL-NPs in ophthalmic TL-NPs formulations. **(B–E)** Particle size frequencies of TL in the reservoir chamber of a methacrylate cell containing ophthalmic TL-NPs formulations [nTL **(B)**, nTL-LMC **(C)**, nTL-MMC **(D)**, nTL-HMC **(E)**]. *n* = 8. The compositions of the ophthalmic TL-NPs formulations are shown in Table 1. *P < 0.05 vs. nTL for each category. The TL-NPs shift to reservoir chamber from the donor chamber. The diffusion of TL-NPs decreased with MC content.

**FIGURE 5:**
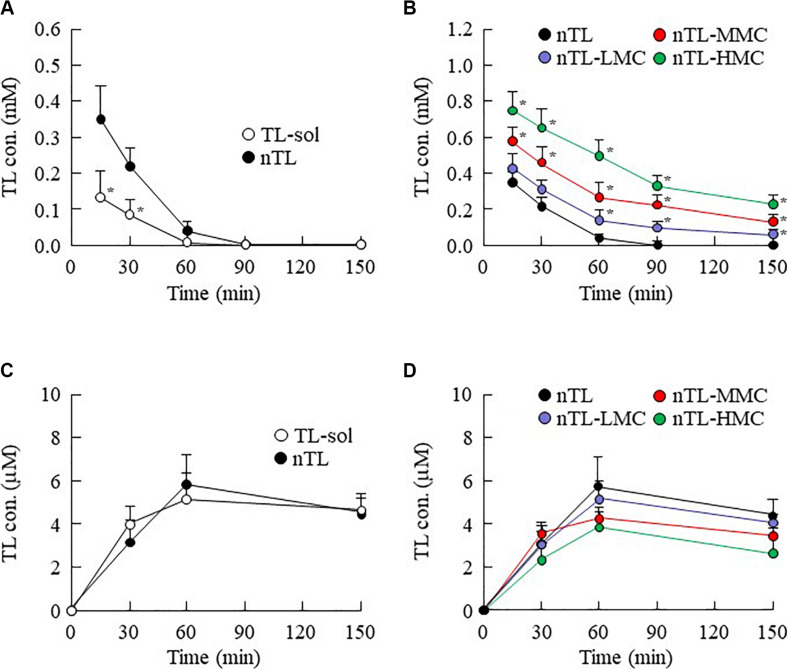
TL contents in the lacrimal fluid and blood of rats instilled with ophthalmic TL-NPs formulations. **(A,C)** TL contents in the lacrimal fluid **(A)** and blood **(C)** of rats instilled with TL-sol and nTL. **(B,D)** TL contents in the lacrimal fluid **(B)** and blood **(D)** of rats instilled with ophthalmic TL-NPs formulations. *n* = 5–8. The compositions of the ophthalmic TL-NPs formulations are shown in Table 1. *P < 0.05 vs. nTL for each category. The TL content in the lacrimal fluid was enhanced with MC content in rats instilled with ophthalmic TL-NPs formulations. Moreover, the addition of MC to the ophthalmic TL-NPs formulations tended to decrease TL levels in the blood after instillation.

**FIGURE 6:**
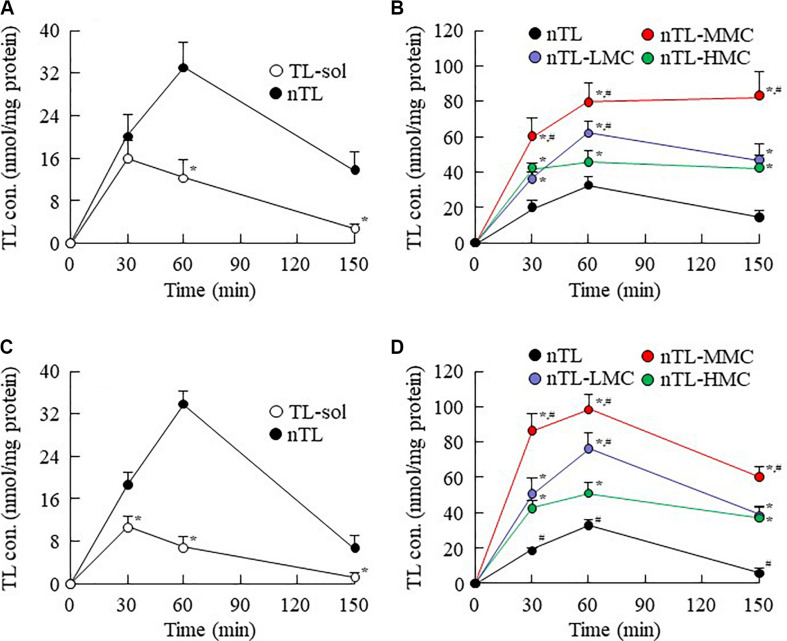
Changes in TL contents in the cornea and conjunctiva of rats instilled with ophthalmic TL-NPs formulations. **(A,C)** TL levels in the cornea **(A)** and conjunctiva **(C)** of rats instilled with TL-sol and nTL. **(B,D)** TL levels in the cornea **(B)** and conjunctiva **(D)** of rats instilled with ophthalmic TL-NPs formulations. *n* = 5–8. The compositions of the ophthalmic TL-NPs formulations are shown in Table 1. *P < 0.05 vs. nTL for each category. ^#^*P* < 0.05 vs. nTL-HMC for each category. The addition of optimum MC (0.5–1.5%) enhanced the TL contents in the cornea and conjunctiva of rats instilled with ophthalmic TL-NPs formulations. On the other hand, excessive MC (3%) prevented the uptake of TL-NPs after instillation.

The changes in viscosity affect the diffusion of TL-NPs in dispersion medium. Therefore, we investigated the relationship between viscosity and diffusion in the ophthalmic TL-NPs formulations. Figure 4 shows the changes in diffusion of TL-NPs in ophthalmic TL-NPs formulations using a methacrylate cell. Almost of all of the TL-NPs that shifted into the reservoir chamber remained in the nano-size range, however, the time needed for TL transfer to the reservoir side from the donor side was increased by increasing the MC content. Figures 5, 6 show the TL contents in the lacrimal fluid (Figures 5A,B), blood (Figures 5C,D), cornea (Figures 6A,B), and conjunctiva (Figures 6C,D) of rats after the instillation of ophthalmic TL-NPs formulations. Although the plasma TL contents were similar between TL-sol and nTL, the TL contents in the lacrimal fluid, cornea and conjunctiva of rats instilled with nTL were significantly higher than those of rats instilled with TL-sol. Otherwise, the addition of low (0.5%) or medium (1.5%) amounts of MC to the ophthalmic TL-NPs formulations tended to decrease the TL content in the blood after instillation while enhancing the TL contents in the lacrimal fluid, cornea and conjunctiva. Although the high (3%) concentration of MC also prolonged TL residence time in the lacrimal fluid after the instillation of TL-NPs, and the TL content in the blood tended to be lower than for TL-NPs containing 0.5% (nTL-LMC) or 1.5% MC (nTL-MMC), the TL contents in the cornea and conjunctiva of rats instilled were significantly lower in comparison with TL-NPs containing 1.5% MC (nTL-MMC). On the other hand, it is important to evaluate the corneal toxicity of ophthalmic TL-NPs formulations. In this study, it was examined whether the cornea of rabbit was damaged by repetitive instillation of nTL, nTL-LMC, nTL-MMC, or nTL-HMC for 1 month (twice a day). As a result, no corneal injury due to instillation was observed in any of ophthalmic TL-NPs formulations.

### Therapeutic Effect on Inflammation in the Conjunctiva by the Instillation of Ophthalmic TL-NPs Formulations

**FIGURE 7:**
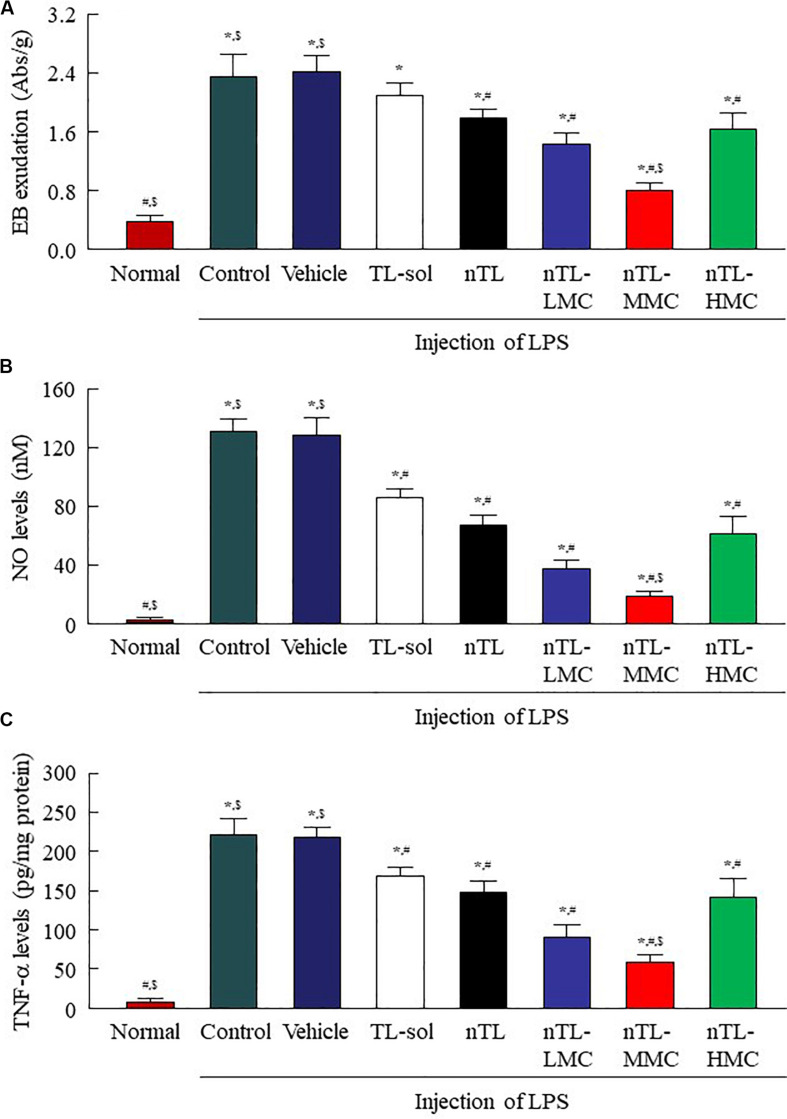
Changes in EB exudation, NO levels and TNF-α levels in the conjunctiva of conjunctivitis rats instilled with ophthalmic TL-NPs formulations. **(A–C)** EB exudation **(A)**, NO levels **(B)**, and TNF-α levels **(C)** in the conjunctiva of conjunctivitis rats 150 min after the instillation of ophthalmic TL-NPs formulations. *n* = 5–8. The compositions of ophthalmic TL-NPs formulations are shown in Table 1. *P < 0.05 vs. normal for each category. ^#^*P* < 0.05 vs. for vehicle for each category. ^$^*P* < 0.05 vs. for nTL-HMC for each category. EB exudation, NO levels and TNF-α levels were enhanced by LPS injection, and these changes were prevented by the instillation of TL. Although the addition of optimum MC (0.5–1.5%) enhanced the preventive effect of TL, the efficacy with excessive MC (3%) was lower than that with 0.5% MC.

In Figures 4–6, we showed that the addition of 0.5 or 1.5% MC to the ophthalmic TL-NPs formulations prolongs drug residence time on the ocular surface, and increases TL uptake into cornea and conjunctiva. Figure 7 shows EB exudation data, and the NO and TNF-α levels in the conjunctiva of conjunctivitis rats instilled with ophthalmic TL-NPs formulations as evidence of the anti-inflammatory effects. The injection of LPS induced EB exudation, and increased the NO and TNF-α levels in the conjunctiva, and the instillation of TL-sol prevented these changes. No significant difference was observed between TL-sol and nTL in EB exudation, NO levels or TNF-α levels in the conjunctiva. On the other hand, the addition of 0.5 and 1.5% MC enhanced the anti-inflammatory effect of the instillation of TL-NPs, and nTL-MMC instillation significantly attenuated EB exudation, and reduced the levels of NO and TNF-α in comparison with TL-sol, nTL, and nTL-LMC. In addition, EB exudation, NO levels and TNF-α levels in the conjunctiva of rats instilled with TL-NPs containing 3% MC (nTL-HMC) were all higher than in rats instilled with TL-NPs containing 1.5% MC (nTL-MMC).

## Discussion

For effective therapy for eye diseases, such as conjunctivitis, it is important to prolong the pre-corneal contact time of ocular drugs. To this end, various ocular drug delivery systems, such as nanosuspensions, nanostructured lipid carriers, liposomes, preformed gels, and bioadhesive polymers have been reported (Almeida et al., 2014; Silva et al., 2017; Grimaudo et al., 2019). We also developed formulations based on HPβCD-coated NPs, and found that these NPs show high rates of uptake into cells *via* energy-dependent endocytosis pathways (Nagai et al., 2014b, 2019). In this study, we aimed to design an *in situ* gel incorporating HPβCD-coated NPs to ensure long residence time of the drug on the ocular surface using TL. Moreover, we demonstrate that the combination of an *in situ* gelling system and NPs enhances the anti-inflammatory effect of TL in comparison with commercially available eye drops and previous TL-NPs formulations without an *in situ* gel system.

Traditional TL eye drops (TL-sol) have been shown to exert various anti-inflammatory actions in experimental studies (Itoh et al., 1993), and are widely used to treat allergic conjunctivitis. Moreover, TL-sol provides treatment for allergic conjunctivitis with negligible toxic effects. Using a bead mill to prepare NPs from TL is relatively easy in comparison with other low solubility drugs such as indomethacin (Nagai et al., 2014a, 2019) or ketoprofen (Nagai et al., 2015, 2018). Because of this, we selected TL to evaluate an *in situ* gel incorporating NPs in this study. Similar to our previous research, the particle size of TL was reduced to the nano range by bead mill treatment (Figures 1A,B), while its crystal structure was retained (Figures 1C,D). Even though the crystal structure is retained, the solubility is enhanced by bead mill treatment (Figure 2A). These results suggest that the solubility of TL increases because of the reduction in the particle size to the nano range. Moreover, the reduction of the particle size to the nano range also enhances the TL/HPβCD inclusion complex (Figure 2A). The TL NPs may be easily included in HPβCD due to their size characteristics in comparison with MPs, resulting in enhancement of the TL/HPβCD inclusion complex. On the other hand, it is known that the cohesion of nanoparticulate solids can be prevented by adsorption to the surface of HPβCD (Mori et al., 2009). Therefore, we measured the dispersibility of HPβCD-coated NPs (nTL), and found that nTL is relatively stable, and ~71% of TL dispersed 3 months after preparation (Figure 3D).

Ophthalmic *in situ* gelling systems have been designed to prolong the pre-corneal contact time of ophthalmic formulations. Therefore, we attempted to prepare *in situ* gels incorporating HPβCD-coated NPs (nTL-LMC, nTL-MMC, and nTL-HMC), and evaluate the effect of MC addition on the gel characteristics. Although the solubility, zeta potential, particle size, and nanoparticle number for TL in the ophthalmic formulations were similar with or without MC, the dispersibility was enhanced by MC with no aggregation or precipitation observed for 3 months (Figures 2, 3). Viscosity is the main factor for increasing the dispersibility of particles, and viscosity was found to be enhanced by the addition of MC (Figure 2). Therefore, enhanced viscosity may increase the dispersibility in ophthalmic TL-NPs formulations. Otherwise, the solubilized TL accounts for ~2% of the total TL in all formulations (the remaining ~98% exists as solid TL nanoparticles, Figure 2B). This result showed that almost no effect of dissolved fraction of TL was observed in this assay.

**FIGURE 8:**
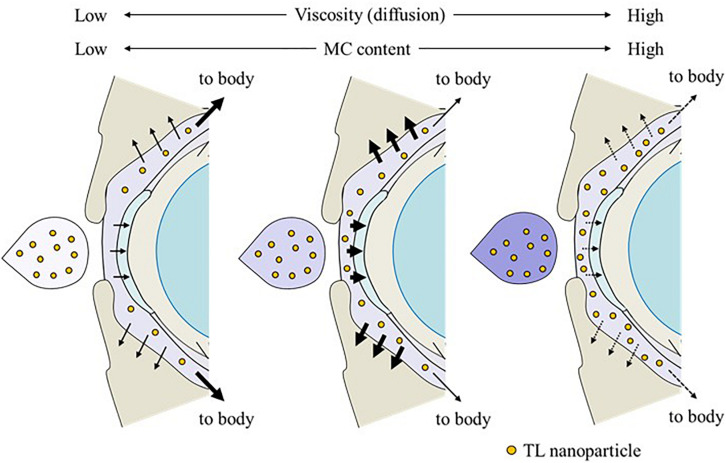
Effect of the MC content on TL behavior on the ocular surface of rats instilled with ophthalmic ISG-formulations containing TL-NPs.

It is thought that changes in viscosity also affect the diffusion behavior of NPs on the ocular surface following instillation. Therefore, we investigated the *in vitro* diffusion behavior of TL-NPs and the *in vivo* accumulation of TL in ocular tissues after instillation. Although TL-NPs shifts to the reservoir chamber from the donor chamber, the TL particles were found to aggregate slightly to a particle size of 80–350 nm (Figure 4). However, this aggregation was lowered by increasing MC content in the ophthalmic TL-NPs formulations (Figures 4B–E). From these results, aggregation might be caused by the instability of the receptor medium (saline) used in the reservoir chamber, and the dilution of the additives (HPβCD and MC) that act to prevent aggregation. In the *in vitro* study, the diffusion behavior of TL-NPs decreased with increasing MC content (Figure 4). The solubility was not changed by the addition of MC (Figure 2B), and the solubility in the nTL, nTL-LMC, nTL-MMC, and nTL-HMC were similar. Therefore, it was suggested that the changes in diffusion behavior of TL-NPs was not affected by the solubility. On the other hand, the experiments for diffusion behavior (Figure 4) were performed at 37°C with gelling of MC, and almost of all of the TL-NPs that shifted into the reservoir chamber remained in the nano-size range. These results suggested that the enhanced viscosity in the MC gelling base prevented the release rate of TL-NPs, resulting in the decrease in diffusion behavior of TL-NPs. In the *in vivo* study, the TL content in the ocular tissues was found to increase with increasing MC content in the ophthalmic TL-NPs formulations, while the addition of MC led to a decreased TL content in the blood (Figure 5). Moreover, the addition of 0.5–1.5% MC enhanced the TL content in the cornea and conjunctiva (Figure 6), while 3% MC prevented TL-NPs uptake after instillation. The TL content in the blood was increased by the absorption from intestine through the nasolacrimal drainage. Although, in the TL-NPs with high MC, the drug supply to the intestine is continuous, the temporary drug supply is lower than TL-NPs with low MC, since the high MC in the ophthalmic TL-NPs formulations prolongs drug residence time on the ocular surface. The drugs levels in the intestine through the nasolacrimal drainage may be related the decreased TL content in the blood. Taken together, we hypothesize that enhanced viscosity prolongs the pre-corneal and pre-conjunctival contact time of TL-NPs. Otherwise, the diffusion of TL-NPs into the lacrimal fluid is low, resulting in some extent of viscosity increased the drug uptake in the cornea and conjunctiva (Figure 8). Further studies using rabbits and monkeys are needed to clarify the usefulness of *in situ* gelling systems based on MC and TL-NPs, because rats and humans show differences in the organizational structure and drug behavior in the eye.

Furthermore, we also demonstrated that ophthalmic ISG-formulations of TL-NPs suppress conjunctival inflammation. Conjunctivitis is a collective term for diseases involving inflammation of the conjunctiva, and are classified as acute and chronic according to the severity of the clinical response and type of onset (Azari and Barney, 2013; American Academy of Ophthalmology, 2018). Inflammation in the conjunctiva can be induced by the injection of LPS, a major component of the outer cell wall of gram-negative bacteria, and the LPS-induced inflammation model has been used to evaluate anti-inflammation effects (Shapira et al., 1996; Allon et al., 2010). Shapira et al. (1996) and Allon et al. (2010) reported that LPS is a potent simulator of monocyte and macrophage cytokine secretion, and that the inflammation induced by LPS also induces a variety of inflammatory mediators, such as NO and TNF-α, both of which are known to play important roles in acute and chronic inflammatory processes. In addition, the inflammation resulting from the injection of LPS induces vessel leakage (Shapira et al., 1996; Allon et al., 2010). In this study, EB exudation, NO levels and TNF-α levels were all enhanced by the injection of LPS (Figure 7), but were prevented by the instillation of TL, and the preventive effect of nTL tended to be higher than that of TL-sol. Also, the addition of 0.5 or 1.5% MC enhanced the preventive effect of TL-NPs. However, the preventive effect of the TL-NPs formulation containing 3% MC was lower than that the TL-NPs containing 0.5 or 1.5% MC (Figure 7). These results support the data for the TL contents in the ocular surface after the instillation of ophthalmic TL-NPs formulations (Figures 5, 6).

In conclusion, we succeeded in preparing ophthalmic ISG-formulations with TL-NPs, and found that *in situ* gelling systems based on MC and TL-NPs increase the ocular residence time and drug absorption into the cornea and conjunctiva. It seems that TL-NPs with an optimum amount of MC results in higher TL contents in the cornea and conjunctiva after instillation in comparison with TL-NPs with excessive MC, probably result of the balance between higher residence time and faster diffusion of TL-NPs on the ocular surface. We found that the optimum level of MC (type SM-4) was 0.5–1.5% in ophthalmic ISG-formulations with NPs. These findings provide significant information that can be used to design further studies aimed at developing ophthalmic nanomedicines.

## Data Availability Statement

The raw data supporting the conclusions of this article will be made available by the authors, without undue reservation, to any qualified researcher.

## Ethics Statement

The animal study was reviewed and approved by the Pharmacy Committee for Animal Research at Kindai University (1 April 2013, project identification code KAPS-25-003).

## Author Contributions

NN conceived and designed the study and wrote the manuscript. NN and MM performed the experiments for the formulation and evaluation of nanoparticles, and analyzed the data. SD and HO performed the drug behavior experiments using rats. SD, HS, and NY performed the experiments to test the therapeutic effect on inflammation. All authors significantly contributed to the conception and design of the study, and to the interpretation of the data.

## Conflict of Interest

The authors declare that the research was conducted in the absence of any commercial or financial relationships that could be construed as a potential conflict of interest.
